# Shoulder Tendon Adaptations Following a Graded Exercise Test to Exhaustion in Highly Trained Wheelchair Rugby Athletes With Different Impairments

**DOI:** 10.3389/fresc.2021.755466

**Published:** 2022-01-18

**Authors:** Fransiska Marie Bossuyt, Barry S. Mason, Simon Briley, Thomas J. O'Brien, Michael L. Boninger, Ursina Arnet, Victoria Louise Goosey-Tolfrey

**Affiliations:** ^1^Shoulder, Health and Mobility Group, Swiss Paraplegic Research, Nottwil, Switzerland; ^2^Human Performance Laboratory, Faculty of Kinesiology, University of Calgary, Calgary, AB, Canada; ^3^Peter Harrison Centre for Disability Sport, School of Sport, Exercise, and Health Sciences, Loughborough University, Loughborough, United Kingdom; ^4^Department of Physical Medicine and Rehabilitation, School of Medicine, University of Pittsburgh, Pittsburgh, PA, United States

**Keywords:** ultrasound, exercise test to exhaustion, para-athlete, supraspinatus tendon, biceps tendon, sports and exercise medicine

## Abstract

**Objective:**

This study aimed to identify acute changes in biceps and supraspinatus tendon characteristics before and after a graded exercise test to exhaustion (GXT) in highly trained wheelchair rugby (WR) athletes. A secondary aspect was to define chronic tendon adaptations related to the impairment of the athlete and the occupation of the tendon within the subacromial space (occupation ratio).

**Methods:**

Twelve WR athletes with different impairments (age = 32 ± 6 years; body mass = 67.2 ± 11.2 kg; 9.0 ± 3.6 years competing) volunteered for this study. Performance Corrected Wheelchair Users Shoulder Pain Index was used to quantify shoulder pain. Quantitative Ultrasound Protocols (QUS) were used to define supraspinatus and biceps tendon thickness, echogenicity, and echogenicity ratio of both dominant and non-dominant shoulder before and after the GXT including 22 ± 3.1 min submaximal propulsion and 10.2 ± 1.7 min maximal propulsion on a treadmill. Furthermore, the acromio-humeral distance (AHD) defined from ultrasound (US) images was used to calculate the occupation ratios.

**Results:**

A mixed-effect multilevel analysis that included shoulder as grouping variable, demonstrated a significant reduction in the echogenicity of the biceps following GXT whilst controlling for impairment [spinal cord injury (SCI) and non-SCI] and the occupation ratio (β = −9.01, SEβ = 2.72, *p* = 0.001, 95% CI = [−14.34; −3.68]). This points toward fluid inflow into the tendon that may be related to overload and acute inflammation. In addition, persons with a SCI (*n* = 8) had a thicker supraspinatus tendon in comparison to persons with non-SCI (*n* = 3) which may be related to chronic tendon adaptations (β = −0.53 mm, SEβ = 0.26, *p* = 0.038, 95% CI = [−1.04; −0.03]). Finally, a greater occupation ratio was associated with signs of tendinopathy (i.e., greater biceps and supraspinatus tendon thickness, and lower supraspinatus echogenicity and echogenicity ratio).

**Conclusion:**

Acute biceps tendon adaptations in response to the GXT in highly trained WR athletes were evident with chronic adaptations in the supraspinatus tendon being related to the impairment of the athlete. Ultrasound can be used to monitor tendon adaptations in WR athletes for medical diagnosis to assist the scheduling and type of training.

## Introduction

Wheelchair rugby (WR) is a fast-paced, paralympic sport played by athletes with a variety of health conditions, with impaired trunk and upper limb function (1). Elite WR players push at high speeds with frequent stops and starts during both competition and training (2, 3). With overhead activities such as passing and catching, also a common feature of WR (4), the demands placed on the shoulder and the potential risk of injury and shoulder pain are likely to be elevated (5, 6). Indeed, 9 out of 12 tetraplegic WR-athletes reported some shoulder pain during activity in the past week (7) and 7 out of 8 elite WR players reported pain after exercise (8). However, the extent of the demands during WR and the risk of pain and pathology in WR athletes remains unclear (9, 10).

Musculoskeletal ultrasound (US) has become a popular tool for identifying musculoskeletal pathologies and monitoring tendon health, due to its low cost, ease of use, and non-invasive approach (11–14). Research using US and MRI has indicated that wheelchair users with a spinal cord injury (SCI) experience a number of shoulder pathologies, with tendinopathies or chronic tendon degeneration of the bicipital and supraspinatus tendons amongst the most common (15–17). Supraspinatus and biceps tendinopathy has also been associated with impingement due to a reduction in the sub-acromial space and therefore a greater occupation ratio [i.e., thickness of the tendon relative to the acromio-humeral distance (AHD)] (18), which naturally occurs during overhead and propulsion activities (18–20). Previous research identified differences in the occupation ratio between persons with subacromial impingement syndrome and healthy controls which further underscored the value of not only investigating tendon thickness and AHD separately (21, 22). Subsequently, ample research has utilized US to establish the thickness and structure of the supraspinatus and biceps tendon, as well as the AHD to quantify the subacromial space, in manual wheelchair users with SCI (20, 23–25). However, previous research has primarily focused on SCI wheelchair users and to date, only one study investigated shoulder tendon characteristics in a sample of WR athletes including persons with a tetraplegia (*n* = 11), paraplegia (*n* = 21), and non-SCI (*n* = 2) (26). While it is valuable to investigate homogeneous samples of persons with similar injuries, the lack of research on wheelchair users with non-SCI impairments causes a gap in the literature.

Monitoring tendon adaptations in response to acute loading is needed to better understand the development of tendon degeneration, and ultimately to be able to intervene and prevent injuries. Previous studies have therefore identified acute tendon adaptations pre- and post-fatiguing wheelchair propulsion performed in the users' daily chair (25, 27). More specifically, a 15-min fatigue protocol in combination with treadmill propulsion at different power outputs, and maximum sprint and strength tests, induced an acute reduction in supraspinatus tendon thickness in a population-based sample of 50 wheelchair users with SCI when controlling for fatigue and subject characteristics (25). However, the 15-min fatigue protocol in itself did not induce significant shoulder tendon changes in 60 wheelchair users of which 80% were athletes (27). Furthermore, a graded treadmill-based propulsion test to maximum exhaustion did not induce significant changes in shoulder tendons in 15 wheelchair users (28). Progressing to wheelchair basketball and WR game play, van Drongelen et al. (26) noted a significant decrease in mean echogenicity ratio of the biceps tendon representing potential fluid inflow into the tendon following these sporting activities. That said, the acute changes following the repetitive activities apparent in these sports differed based on the amount of playing time. Moreover, a lower echogenicity ratio was observed both at the onset and following the competitive games in players who reported shoulder pain.

To date, no study has investigated shoulder tendon adaptations following repetitive activity up to maximum exhaustion, when the musculoskeletal system is unstable and particularly prone to tissue adaptations (29), in highly trained WR athletes with different physical impairments. Subsequently, the aims of the current study were (1) to identify acute changes in biceps and supraspinatus tendon characteristics following a graded exercise test to exhaustion (GXT) in highly trained WR athletes, and (2) to define differences in chronic tendon adaptations related to the impairment of the athlete and the occupation ratio. We thereby also investigated a potential association between changes in shoulder tendon characteristics and shoulder pain.

## Methods

### Participants

Twelve highly trained National level WR players consisting of 11 males and one female player (age = 32 ± 6 years; body mass = 67.2 ± 11.2 kg) provided their informed consent to participate in the current quasi-experimental study with a repeated measures design. The study was approved by the local ethical advisory committee. Participants were grouped according to those who had a tetraplegic (complete lesion level between cervical vertebrae C5 and C7) SCI (*n* = 8) and those who had a non-spinal impairment (cerebral palsy, critical care polyneuropathy, brachial plexus nerve injury, and Roberts syndrome: non-SCI; *n* = 4). All participants with SCI were 17 years or older when they sustained their injury.

### Experimental Design

The assessments are partly included in the annual monitoring programme of the WR athletes and briefly described below. The additional assessments that are included in the annual monitoring programme (e.g., 30 s Wingate test on a dual roller wheelchair ergometer aimed to determine anaerobic capacity) will be presented elsewhere. Body mass and mass of the daily and rugby chair were obtained to the nearest 0.1 kg with seated balance scales (Seca, Birmingham, UK). Participants completed the Wheelchair Users Shoulder Pain Index (WUSPI), with a performance-corrected version (PC-WUSPI) used to indicate the magnitude of shoulder pain (30, 31). The Upper Extremity Pain Symptom Questionnaire (PSQ) was used as an auxiliary questionnaire to the PC-WUSPI to identify the presence of pain and establish whether shoulder pain was unilateral or bilateral (32). The continuous GXT was performed in participants customized rugby wheelchairs (Rugby chair mass: 17.0 ± 1.4 kg, handrim diameter: 0.54 ± 0.01 m, chamber: 18.1 ± 1.8°) on a motor driven treadmill (HP Cosmos, Traunstein, Germany). Musculoskeletal US examinations were taken to determine (2) the characteristics of the supraspinatus and biceps tendons of both dominant and non-dominant side pre- and post- the GXT, and (3) the AHD pre- the GXT.

### Ultrasound

**Figure 1:**
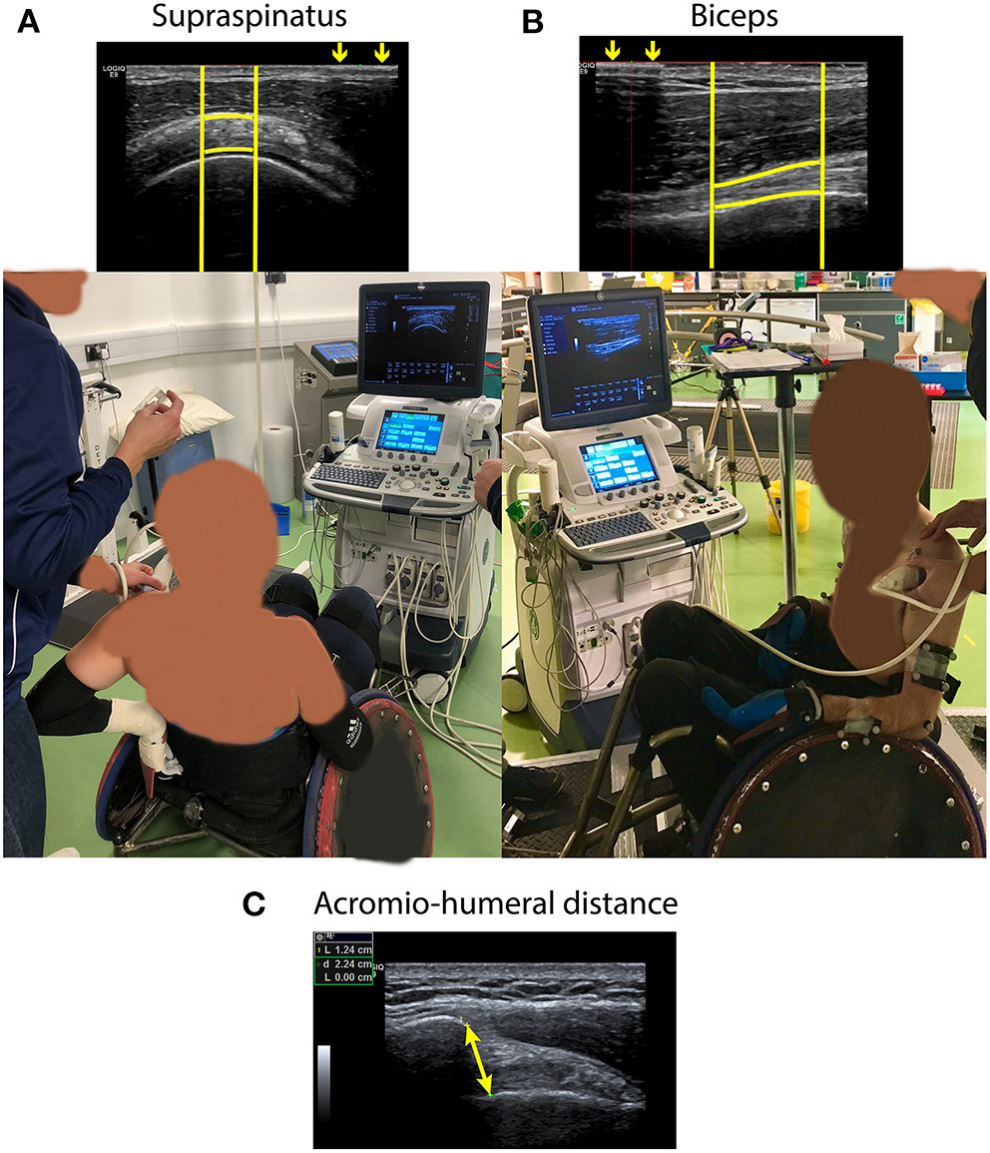
**(A)** Ultrasound image of supraspinatus tendon taken following Quantitative Ultrasound Protocols (QUS) and position in which the image was taken. **(B)** Ultrasound image of biceps tendon taken following QUS and position in which the image was taken. For both **(A)** and **(B)**, arrows demonstrate the interference pattern that resulted from a metal marker taped to the skin from which the bright vertical lines were defined. Bright horizontal lines following the tendon borders are manually defined. **(C)** Ultrasound image of the acromio-humeral distance (i.e., the shortest distance between the anterior inferior edge of the acromion and the humeral head). The image was taken in the same position as the image of the biceps tendon, but with the thumbs facing upwards.

Two images of the biceps and supraspinatus tendon of both the dominant and non-dominant side were taken in a randomized order at two different time points following previously validated Quantitative Ultrasound Protocols (QUS) (13, 14) with an US device (Legic E9, GE Healthcare, USA). Quantitative Ultrasound Protocols has been used previously before and after fatiguing tasks (25–27) and allows limited error in probe location between measuring time points because of the use of a steel marker taped to the skin that allows to identify the region of interest (ROI). The QUS images were taken before any tasks took place (duration ca. 30 min) (pre-exercise; time point 0) and after the GXT (duration ca. 15 min) (post-exercise; time point ~ 1 h 45 min). For the longitudinal images of the biceps tendon, participants were seated in their rugby wheelchairs with their arms at 0° abduction and 90° elbow flexion with the palm facing upwards (Figure 1B) (13, 14, 25). To take transverse images of the supraspinatus tendon, the participants were asked to externally rotate the shoulder and place the palm flat on the back of the wheelchair (Figure 1A) (13, 14, 25). Additionally, three images of the AHD were taken pre-exercise in a seated position with the arms at 0° abduction and 90° elbow flexion with the thumbs facing upwards (Figure 1C) (33).

All US images were analyzed in a randomized order by the same examiner requiring 20 min per image (FMB). Using the ROI, tendon images were analyzed to calculate tendon thickness (mean distance between horizontal tendon borders), echogenicity (mean grayscale of the ROI), and echogenicity ratio (echogenicity relative to the mean grayscale of the muscle above the tendon). The shortest distance between the anterior, inferior edge of the acromion and the head of the humerus was used to define AHD. The mean of each repeated variable at the respective time point was used for further analysis. Tendon occupation ratios were calculated as a percentage of the tendon thickness relative to the AHD.

### Graded Exercise Test to Exhaustion

Immediately after the pre-exercise US measures, participants completed a 10 min self-selected warm-up in their own rugby wheelchairs at speeds lower than the subsequent incremental exercise test. Following 5 min passive rest, participants completed a submaximal incremental exercise test. In brief, participants completed 3-min blocks of exercise, where speed was increased by 0.2 m/s (m.s^−1^) for low functioning participants [World WR (1) classification <2.0] or 0.3 m.s^−1^ for higher functioning participants (WWR classification ≥2.0) for determination of speed at blood lactate threshold. The starting speed was individualized according to functional capacity (SCI or non-SCI), WWR classification, and previous test results (where available), with the goal to obtain similar total test durations for all participants (34). Termination of the submaximal test occurred when blood lactate concentration exceeded 4 mmol/l and/or a Rate of Perceived Exertion (RPE) of 17 was reached (34, 35). One investigator (TJO'B) gave all verbal encouragement which included specific quotes such as “Come on, keep pushing,” “Keep pushing all the way to the end,” “You're doing great, maximum effort,” with these quotes kept consistent between participants. Following ~30 min passive rest, participants completed the GXT with speed increments of 0.1 ms^−1^ every minute to determine maximal oxygen uptake (*V*O_2peak_) (Metalyzer® 3B, Cortex Biophysik GmbH, Leipzig, Germany) (34). Starting speed for this test was based on visual determination of their blood lactate threshold from the submaximal test. Strong verbal encouragement was given throughout until they could not maintain the speed of the treadmill, which terminated the test. Following completion of the exercise protocol, post-exercise US measurements were completed.

### Statistical Analysis

Statistical analyses were conducted with STATA software (version 14, StatCorp, LP, College Station TX, USA). Subject characteristics between SCI and non-SCI participants were compared using independent sample *t*-tests. The intraclass correlation (ICC) of the repeated US measures (i.e., at each time point of data collection, we collected two images of the biceps tendon and two images of the supraspinatus tendon) were calculated with a two-way random effects model (absolute agreement, random effects: participant ID and measure) to confirm good reliability between measurements at a single time point (36). Ultrasound measures with a poor reliability (ICC ≤ 0.5) would be removed from further analyses (36). Ultrasound data from all shoulders were included into a mixed-effect multilevel analysis to identify the association between dependent variables (tendon characteristic) and time point (pre- or post-exercise; acute adaptations), whilst controlling for impairment (SCI and non-SCI; chronic adaptations) and the occupation ratio. Shoulder (dominant or non-dominant) was included as a grouping variable (random intercept). Normality of the residuals was confirmed with Histogram, Quantile normal plots, and Shapiro-Francia tests. Likelihood-ratio tests after estimation of the unadjusted and adjusted model were used to confirm the significance of the random intercept. Pearson's correlations were used to explore the relationship between shoulder pain (PSQ scores) and tendon characteristics from all shoulders pre- and post-exercise. Correlations were described as negligible (<0.3), low (0.3–0.5), moderate (0.5–0.7), and high (>0.7) (37). Statistical significance was accepted as *p* < 0.05.

## Results

The physical characteristics of participants are presented in Table 1. No significant difference existed between SCI and non-SCI except that persons with SCI had a lower *V*O_2peak_ (*p* = 0.04) and spent less time in the gym (*p* = 0.02).

**Table 1:** Characteristics of participants and their wheelchair (whc) stratified by impairment.

	**Total (*n* = 12)**	**SCI (*n* = 8)**	**Other (*n* = 4)**	***p***
Age (years)	31.8 ± 5.6	31.8 ± 6.3	31.8 ± 4.8	
Height (cm)	170 ± 22.1	177.3 ± 14.1	155.5 ± 30.1	
Body mass (kg)	66.3 ± 12.1	68.4 ± 11.6	62.1 ± 13.9	
Years whc use	14.8 ± 8.3	12.6 ± 6.5	19.3 ± 10.8	
Years competing	9.0 ± 3.6	9.8 ± 4.0	7.5 ± 2.4	
Court hours per week	8.4 ± 2.8	8.1 ± 2.9	9 ± 3.2	
Gym hours per week	4.5 ± 2.3	3.4 ± 1.5	6.5 ± 2.4	**0.02** ^*^
Other sports e.g., swimming, handbike hours per week	2.2 ± 1.8	1.9 ± 1.6	2.8 ± 2.2	
Total training hours per week	15.1 ± 4.9	13.5 ± 3.8	18.3 ± 6.6	
Rugby chair mass (kg)	17.0 ± 1.4	16.7 ± 1.3	17.6 ± 1.4	
Tire pressure (psi)	148 ± 36	161 ± 38	123 ± 36	0.07
VO_2_peak (ml/kg/min)	25.26 ± 7.88	22.08 ± 7.34	31.62 ± 4.60	**0.04** ^*^

*An*

* *and bold values are marked from the independent sample t-tests with significant p-values (α = 0.05)*.

The ICC of the repeated measures ranged between 0.71 and 0.99 representing high correlations for AHD and all investigated tendon characteristics; except for the post-measurements of the supraspinatus echogenicity on the dominant side (ICC = 0.52) representing a moderate correlation.

### Changes in Tendon Characteristics Following Graded Exercise Test to Exhaustion (Acute Adaptations)

**Figure 2:**
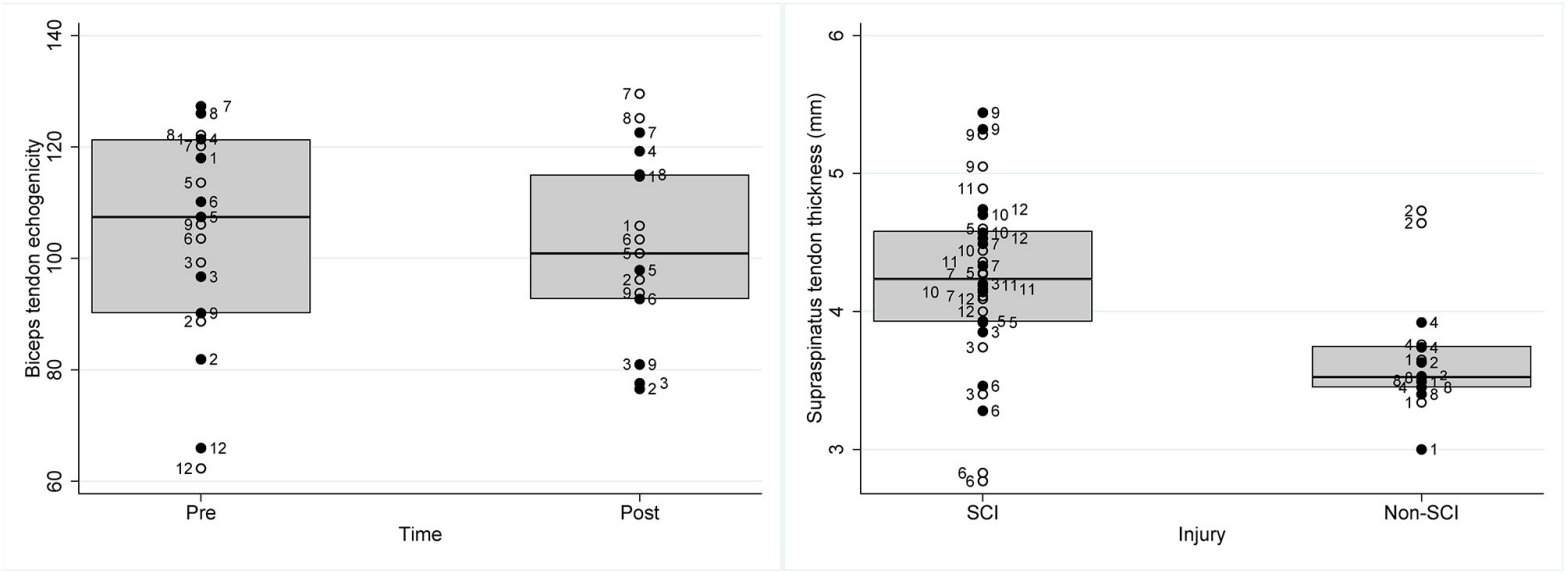
Box plots representing raw data of individual wheelchair rugby athletes of (left) biceps tendon echogenicity pre and post a graded exercise test to exhaustion and (right) supraspinatus tendon thickness (mm) in athletes with SCI and non-SCI. Full circles with participant ID on the right side of the circle represents data of the dominant shoulder of the respective athlete, the empty circles with the participant ID on the left side of the circle represents data of the non-dominant shoulder of the respective athlete.

The only tendon characteristic to significantly change post-exercise was the echogenicity of the biceps tendon which significantly reduced post-exercise (β = −9.01, SEβ = 2.72, p = 0.001, 95% CI = [−14.34; −3.68]) (Table 2; Figure 2). More specifically predictive margins of biceps tendon echogenicity changed from 105.14 before GXT (SE = 5.79, 95% CI = [93.79; 116.49]) to 98.11 following GXT (SE = 5.83, 95% CI [86.69; 109.53]) (p < 0.001). No further adaptations were observed over time.

**Table 2:** Unadjusted characteristics [mean (SD)] of the biceps and supraspinatus tendon pre- and post- a fatiguing bout of exercise, the occupation ratio, and acromio-humeral distance (AHD) in WR players with SCI and Non-SCI.

	**Pre**		**Post**		**Mixed model**		
	**SCI**	**Non-SCI**	**SCI**	**Non-SCI**	**Time**	**SCI**	**Interaction**
					**P-value**	**P-value**	**P-value**
**Acromio-humeral distance (mm)**	11.9 (2.1)	10.6 (1.2)					
**Biceps tendon**							
Thickness (mm)	2.7 (0.5)	3.3 (1.0)	2.7 (0.4)	3.6 (1.0)	0.867	0.766	0.158
Occupation ratio (%)	23.1 (3.2)	32.0 (12.1)					
Echogenicity	100.2 (19.6)	111.3 (18.1)	98.0 (17.3)	107.5 (16.6)	0.001*	0.335	0.222
Echo ratio	2.3 (0.6)	1.9 (0.6)	2.2 (0.6)	2.0 (0.7)	0.607	0.524	0.536
**Supraspinatus tendon**							
Thickness (mm)	4.3 (0.6)	3.6 (0.5)	4.2 (0.7)	3.7 (0.4)	0.387	0.038*	0.219
Occupation-ratio (%)	36.5 (7.05)	34.8 (6.9)					
Echogenicity	98.8 (10.4)	98.2 (9.0)	96.6 (11.4)	93.5 (9.2)	0.323	0.643	0.504
Echo ratio	1.2 (0.2)	1.3 (0.3)	1.3 (0.2)	1.3 (0.5)	0.181	0.808	0.454

Significant p-values (alpha = 0.05) from the mixed-effects multilevel analysis are marked with bold values and an *.

### Association Between Tendon Characteristics, Impairment, and Occupation Ratio (Chronic Adaptations)

Persons with a non-SCI had a thinner supraspinatus tendon (β = −0.53 mm, SEβ = 0.26, *p* = 0.038, 95% CI = [−1.04; −0.03]) in comparison to SCI (Table 2; Figure 2). More specifically, predictive margins for supraspinatus thickness for persons with SCI were 4.19 mm (SE = 0.14, 95% CI = [3.91; 4.47]), while for persons with non-SCI this was 3.74 mm (SE = 0.20, 95% CI = [3.35; 4.14]) (*p* < 0.001). In addition, it was found that biceps and supraspinatus tendon thickness were positively associated with the occupation ratio (biceps thickness: β = 0.07, SEβ = 0.01, *p* < 0.001, 95% CI = [0.05; 0.09]; supraspinatus thickness: β = 0.06, SEβ = 0.01, *p* < 0.001, 95% CI = [0.04; 0.08]). Alternatively, for the supraspinatus, echogenicity (β = −0.78, SEβ = 0.21, *p* < 0.001, 95% CI = [−1.19; −0.37]), and echogenicity ratio (β = −0.02, SEβ = 0.01, *p* = 0.002, 95% CI = [−0.03; −0.01]), were negatively associated with the occupation ratio.

### Shoulder Pain

Of the 12 participants, seven reported no shoulder pain (PC-WUSPI mean = 0.07 ± 0.19), whereas five reported shoulder pain (PC-WUSPI mean = 15.5 ± 14.0; range 7.9–40.4). Of these participants, two experienced unilateral and three experienced bilateral shoulder pain. Furthermore, the group of participants with shoulder pain consisted of three persons with SCI (PC-WUSPI mean = 20.5 ± 17.2; range 10.3–40.4), and two persons with a non-SCI (PC-WUSPI mean = 7.8 ± 0.1; range: 7.8–7.9). No relationships between tendon characteristics and shoulder pain were observed pre-exercise (Table 3). Post-exercise it was revealed that decreased echogenicity of the supraspinatus tendon was correlated with increased pain.

**Table 3:** Association between acromio-humeral distance (AHD), tendon characteristics, and shoulder pain.

	**Pre**		**Post**	
	***r***	***p***	***r***	***p***
**AHD (mm)**	0.311	0.139		
**Biceps tendon**				
Thickness (mm)	–0.205	0.400	–0.205	0.400
Occ ratio (%)	–0.315	0.189		
Echogenicity	–0.043	0.861	0.151	0.563
Echo ratio	0.335	0.160	0.035	0.889
**Supraspinatus tendon**				
Thickness (mm)	0.055	0.799	–0.112	0.602
Occ ratio (%)	–0.196	0.359		
Echogenicity	–0.279	0.186	–**0.434**	0.034^*^
Echo ratio	–0.115	0.594	–0.022	0.918

*An*

* *and bold values are marked from the Pearson's correlations with significant p-values (α = 0.05)*.

## Discussion

This novel study demonstrated significant adaptations in tendon characteristics in 12 highly trained WR athletes with different impairments. Acute adaptations were demonstrated in the reduction in the echogenicity of the biceps tendon immediately following the GXT, pointing toward fluid inflow into the tendon (darker tendon). Chronic tendon adaptations are associated with the impairment of the athlete, athletes with SCI presented significantly thicker supraspinatus tendon as compared to athletes with non-SCI. In addition, a greater occupation ratio was positively associated with signs of chronic tendon degeneration. Finally, shoulder pain was only associated to supraspinatus echogenicity following the GXT. Overall, these findings are in line with the high metabolic activity of human tendons (38).

### Acute Tendon Adaptations

The significant acute reduction in the echogenicity of the biceps tendon following the GXT may be related to acute overload and inflammation referred to as reactive tendinopathy which differs from normal tendon adaptation to tensile load (39). With insufficient time to recover, such acute overload, can lead to chronic tendon degeneration or degenerative tendinopathy (39, 40).

In accordance with the present results, an earlier study that employed the same QUS demonstrated a reduction in echogenicity ratio of the biceps tendon in response to a competitive wheelchair basketball or WR game (26). Importantly, however, although the current study reported a reduction in the echogenicity, there was no change in the echogenicity ratio. This suggests that there was a reduction in both the grayscale of the biceps tendon and the muscle above the tendon and may be caused by an overall fluid shift to the arm impacting both muscle and tendon simultaneously. This on its turn, would be related to fluid mobilization rather than inflammation. As a result of the GXT, different changes in the muscle above the tendon may have occurred in the present study due to the potential for more rest in between propulsion bouts, and different movements when compared to actual game play (E.G. turning and ball handling) (26). Nevertheless, both studies support the notion that there are acute adaptations in the biceps tendon grayscale following straining propulsion in WR athletes that could play a role in the development of shoulder pathology and pain in this population.

In line with the work of van Drongelen et al. (26), we did not demonstrate an increase in biceps tendon thickness following the GXT which is expected to coincide with acute overload. The current study investigated changes in the biceps and supraspinatus tendon in response to exercise including 22 ± 3.1 min submaximal propulsion and 10.2 ± 1.7 min maximal propulsion, and the study of van Drongelen et al. (26) investigated changes following game play varying between 10 and 70 min depending on the participants time on court. The duration of the activities in both studies may not have been long enough to induce an increase in tendon thickness. Like wheelchair propulsion, swimming is a repetitive sport that places great demands on the shoulder tendon structures while the AHD is reduced. To this effect, an acute increase in supraspinatus tendon thickness has been reported immediately post a high intensity swim training (3.5 km in 2 h) with smaller, but still significant increases in thickness in response to high volume swim trainings (7 km in 2 h) in eight state and national level swim athletes (41). Further research is needed to determine a potential increase in biceps tendon thickness with longer bouts of intense propulsion activity. Nevertheless, findings of this study support the added value of investigating the gray-scale of the tendon, which may be more sensitive to acute changes in reactive tendinopathy, rather than focussing on changes in tendon thickness only.

In contrast to earlier findings (25), this study did not present acute changes in the supraspinatus tendon following repetitive propulsion activity. More specifically, 15-min maximum voluntary propulsion resulted in a significant reduction in supraspinatus tendon thickness (25). A reduction in tendon thickness, a typical response to tensile loading, can be related to alignment of the tendon collagen fibers in the direction of the applied stress (42). A possible explanation for the different results may be related to the higher and complete lesion level of the persons with SCI in the current study [tetraplegia, 100% complete injury; (25): paraplegia, 78% incomplete], and GXT, which is likely to cause greater loads on the shoulder muscles and tendons and subsequently result in a different tendon response. In addition, the small sample size [*n* = 12; (25): *n* = 50] should be acknowledged and differences in wheelchair characteristics of the rugby and daily chair [rugby chair mass: 17.0 ± 1.4 kg with camber: 18.1 ± 1.8° vs. daily chair mass of Bossuyt et al. (25): 14.5 ± 2.1 kg with camber 0°], and subsequent altered position are likely to place different demands on the shoulder tendons. For example, fatiguing propulsion in wheelchair users' daily chair caused greatest signs of neuromuscular fatigue in the pectoralis, deltoideus, and upper trapezius (43) while current results and those of van Drongelen et al. (26) suggest that fatiguing propulsion in the rugby chair places greater demands on the biceps brachii tendon. This underlines the importance of the task-dependency of musculoskeletal loading and subsequent tendon adaptations.

### Chronic Tendon Adaptations

This study demonstrated that WR athletes with SCI had a thicker supraspinatus tendon in comparison to WR athletes with a different impairment. Increased tendon thickness may relate to chronic adaptations that causes tendon hypertrophy and strengthened the tendon by increasing its stiffness (12), or may be caused by chronic inflammation and indicate the presence of pathology (39). Interestingly, persons with SCI spent a significantly lower amount of time in the gym compared to non-SCI. With the lack of trunk function in the SCI group compared to the non-SCI group, it may be plausible that daily tasks (such as propelling and transferring into their wheelchair) may further increase the loads on shoulder tendons in SCI. Thus, despite the reduced gym exposure, hypertrophic adaptations may persist. Interestingly, values for the supraspinatus tendon thickness of persons with a tetraplegia in the current study remain lower as compared to those reported previously in a sample of persons with a paraplegia (25). Persons with SCI also had a significantly lower *V°*O_2peak_ further demonstrating differences in functioning between the two groups. Therefore, the previously established differences in volume of activity during rugby games based on functioning of the athletes may play a role in the different tendon adaptations (2).

While the percentage of athletes with shoulder pain in both groups was not markedly different (SCI: 3/8 athletes with pain, non-SCI: 2/4 athletes with pain) the average PC-WUSPI was higher in the SCI group than in the non-SCI group, PC-WUSPI remained below a score of 10. This could also be related to the lack of trunk support in the persons with a tetraplegic SCI thereby increasing loads on the shoulder. However, none of the US measures pre the GXT correlated with pain in the WR athletes which may be due to the small sample size in this study as this reduces the power of the study and increases the risk of type II error. A positive correlation between supraspinatus tendon thickness (defined with QUS) and supraspinatus pathology [Ultrasound Shoulder Pathology Rating Scale (USPRS)] has been established in wheelchair users with SCI (14). However, as far as we are concerned, no previous study compared tendon characteristics in wheelchair athletes with different impairments. In order to better understand the presented chronic adaptations, further imaging is needed to identify potential differences in tendon stiffness, and or inflammatory markers between wheelchair users with SCI and non-SCI impairments. The current findings demonstrate the need for an individualized approach and differentiation between impairments when monitoring tendon adaptations.

A greater occupation ratio for the biceps and supraspinatus was consistently associated with tendon characteristics that have been correlated with increased signs of tendinopathy in wheelchair users with SCI via the USPRS tendon grade (i.e., greater biceps and supraspinatus tendon thickness, and lower supraspinatus echogenicity and echogenicity ratio) (14). Interestingly, the occupation ratio of the supraspinatus remains smaller as compared to able-bodied persons with and without subacromial impingement syndrome (21, 22). It should be considered that the measures used to calculate the occupation ratio (i.e., supraspinatus and biceps tendon thickness and AHD) were taken from different US images with a different position for the supraspinatus tendon thickness. Nevertheless, our results are in line with previous studies that reported a greater occupation ratio in persons with subacromial impingement syndrome vs. healthy controls (21, 22) and support that a smaller space between the tendon and the acromion, or a greater occupation ratio, may be related to signs of chronic tendon degeneration. Therefore, the occupation ratio could be an interesting measure to include in the yearly screening of WR athletes.

## Limitations and Future Directions

While a strength of this study was that our WR players had similar training histories and measures were taken at the same time-point within their training program, the small sample and heterogeneous nature of their injuries and functional capacities limits the generalizability of our findings. More specifically, the small sample size reduces the power of our study and increases the risk of type II errors. To account for the applied nature of this study, we chose a method that was easy and low-cost so it could be included in the monitoring program of WR athletes in the future. That said, it must be noted that the US images are limited in resolution and only allow two-dimensional measurements. Furthermore, it is important to acknowledge that without prior US imaging experience, it does require time and effort (~25 h) to become proficient in taking and analyzing images following the QUS. The use of US elastography, a promising tool to define mechanical properties of the tendon including tendon stiffness, could have provided a more comprehensive understanding of the presented tendon adaptations. We are aware that we did not include a matched-control group, yet this may have been difficult due to the aforementioned heterogenous sample. Differences in hydration level could have impacted the US images and our overall results. Although we did not quantify the hydration level of each individual participant, the athletes, who receive educational support in terms of nutrition and hydration, were asked to arrive to the laboratory in a hydrated state. Furthermore, given the design of our study, the athletes acted as their own controls. We feel to advance our current knowledge it would be helpful to include multiple time-points following a rest period to observe changes in tendon adaptations following rest. It could also be of great benefit to include QUS measures to monitor tendon health longitudinally. Such assessments would allow to gain a better understanding of chronic tendon adaptations and asymmetries in WR athletes and the development of chronic degeneration in this population.

## Conclusion

There are acute biceps tendon adaptations in response to a GXT in highly trained WR athletes. The presented chronic tendon adaptations are associated with the impairment of the athlete (SCI vs. non-SCI) and the occupation ratio and may play a role in the high prevalence of shoulder problems in this population. Including such assessment methods in screening of wheelchair athletes may provide further insights into the long-term consequences of the reported changes and allow us to better understand and monitor shoulder health as well as to improve injury prevention.

## Data Availability Statement

The datasets presented in this article are not readily available because the participants did not consent to sharing their data when they entered the study. Requests to access the datasets should be directed to fransiska.bossuyt@paraplegie.ch.

## Ethics Statement

The studies involving human participants were reviewed and approved by Reference number SSEHS-2626 approved by Regulatory, Compliance and Safety Administrator Loughborough University. The patients/participants provided their written informed consent to participate in this study.

## Author Contributions

FMB, BSM, SB, UA, and VLG-T initiated this study. FMB, BSM, SB, MLB, UA, and VLG-T contributed to the conception and design of the study. FMB, BSM, SB, and TJO'B performed the data collection and were responsible for the data analyses. FMB and BSM performed statistical analyses and drafted the paper. FMB finalized the paper. All authors interpreted the data, critically revised the paper, read, and approved the final paper.

## Funding

This study has been financed by the Peter Harrison Foundation and financial support of the Institute of Advanced Studies (IAS) at Loughborough University. The study was also financed by the Swiss Paraplegic Research supported by the Swiss Paraplegic Foundation.

## Conflict of Interest

The authors declare that the research was conducted in the absence of any commercial or financial relationships that could be construed as a potential conflict of interest.

## Publisher's Note

All claims expressed in this article are solely those of the authors and do not necessarily represent those of their affiliated organizations, or those of the publisher, the editors and the reviewers. Any product that may be evaluated in this article, or claim that may be made by its manufacturer, is not guaranteed or endorsed by the publisher.
